# Effect of Knee Angle, Contractile Activity, and Intensity of Force Production on Vastus Lateralis Stiffness: A Supersonic Shear Wave Elastography Pilot Study †

**DOI:** 10.3390/sports12080211

**Published:** 2024-07-31

**Authors:** Rute Santos, Maria João Valamatos, Pedro Mil-Homens, Paulo A. S. Armada-da-Silva

**Affiliations:** 1 Faculdade de Motricidade Humana, Universidade de Lisboa, 1499-002 Estrada da Costa, Portugal; mjvalamatos@fmh.ulisboa.pt (M.J.V.); pmilhomens@fmh.ulisboa.pt (P.M.-H.); parmada@fmh.ulisboa.pt (P.A.S.A.-d.-S.); 2 Coimbra Health School, Polytechnic University of Coimbra, Rua da Misericórdia, Lagar dos Cortiços, S. Martinho do Bispo, 3045-093 Coimbra, Portugal; 3 H&TRC—Health & Technology Research Center, Coimbra Health School, Polytechnic University of Coimbra, Rua 5 de Outubro, 3045-043 Coimbra, Portugal; 4 CIPER-UC—Interdisciplinary Center for the Study of Human Performance, University of Coimbra, 3004-531 Coimbra, Portugal; 5 Centro para o Estudo da Performance Humana, Faculdade de Motricidade Humana, Universidade de Lisboa, 1499-002 Estrada da Costa, Portugal

**Keywords:** supersonic shear image ultrasound, vastus lateralis muscle, elasticity, elastography, elasticity imaging techniques, ultrasonography

## Abstract

Supersonic shear image (SSI) ultrasound elastography provides a quantitative assessment of tissue stiffness using the velocity of shear waves. SSI’s great potential has allowed researchers in fields like biomechanics and muscle physiology to study the function of complex muscle groups in different conditions. The aim of this study is to use SSI to investigate changes in the stiffness of the vastus lateralis (VL) muscle as a consequence of passive elongation, isometric contraction, and repeated muscle activity. In a single session, 15 volunteers performed a series of isometric, concentric, and eccentric contractions. SSI images were collected from the VL to assess its stiffness before and after the contractions and at various knee angles. Two-way within-subjects ANOVA was used to test the effects of muscle contraction type and knee angle on VL stiffness. Linear regression analysis was employed to assess the relationship between muscle stiffness and the intensity of isometric contractions. After maximal contractions, VL stiffness increased by approximately 10% compared to baseline values, and following maximal isometric (*p* < 0.01) and eccentric contractions (*p* < 0.05). Yet, there was no change in VL shear modulus at the end of concentric contractions. The relaxed VL shear modulus increased with knee flexion both before and after the knee extensor contractions (*p* < 0.001). A linear relationship between the shear modulus and the degree of isometric contraction was observed, although with notable individual variation (R^2^ = 0.125). Maximal contractile activity produces modest increases in relaxed muscle stiffness. The SSI-measured shear modulus increases linearly with the degree of isometric contraction.

## 1. Introduction

The recent developments in ultrasound (US) imaging modalities have enhanced our ability for studying muscle tissue mechanical properties in vivo. This includes the assessment of changes in muscle stiffness associated with stretching and contraction [1,2,3,4,5,6] One such advance is supersonic shear imaging (SSI) [7,8], which allows for the real-time quantification of muscle tissue stiffness in dynamic and relatively unconstrained conditions [9,10,11,12]. The feasibility of SSI to investigate the mechanical properties of the muscle tissue is well documented, including in response to passive muscle elongation [5] and during isometric contractions of different intensities [13]. The good sensitivity of SSI measurements of tissue stiffness has also been explored to address questions related to muscle adaptation to resistance training and stretching [14,15,16,17], muscle co-ordination [18,19], and muscle tissue changes caused by aging [20,21,22], injury, or disease [23]. Compared to other stiffness measurement techniques, such as the use of hardness meters [7,24,25] or torque-angle curves’ determination [17,26], US elastography, in particular SSI, has the additional advantage that it allows for measuring muscle tissue stiffness in several separate regions of muscles and tendons both at rest and during contraction. 

Supersonic shear wave imaging is based on measuring the velocity of propagating mechanical vibrations or shear waves generated by focused US beams. This technique uses acoustic radiation force to generate a series of pushes inside the tissue and ultrafast US acquisition for detecting and measuring the propagation of the induced shear waves [8,27,28]. The generated planar shear wave propagates with a velocity that is directly proportional to the stiffness of the medium. The mathematical relation describing the velocity of the traveling shear waves is then inverted to construct a map of the Young’s modulus [1,12]. The anisotropic character of the muscle tissue influences the propagation velocity of the shear waves and the sensitivity of SSI to measure muscle stiffness. Thus, the velocity of the shear waves during muscle contraction exhibits a direct relationship with the intensity of the contraction only when the shear waves propagate parallel to the length of the muscle fascicles, in other words, when the principal axis of the US probe is oriented parallel to the muscle fibers [1,29,30].

Measuring muscle stiffness is important for understanding muscle function and to monitor muscle status. Several studies have demonstrated the utility of SSI in assessing muscle stiffness across different muscle groups. Good estimates of tibialis anterior stiffness can be obtained with SSI [5]. Stretching has been shown to cause a linear increase in shear modulus in the hamstring muscles [29]. Additionally, in upper extremity muscles, shear wave velocity is linearly related with the degree of isometric submaximal contractions (<60% maximal voluntary contraction (MVC)) [1,31]. This relationship may extend to the full range of isometric torque capacity, particularly in smaller hand muscles [13]. 

Repeated muscle contractions can lead to an increase in muscle stiffness, which may be detected using SSI [12,23,32,33]. Lacourpaille et al. [34] reported increased shear modulus of the elbow flexors 60 min after a series of isokinetic eccentric (Ecc) contractions (three sets of 10 contractions at 120°·s^−1^) [23]. This increase normalized within 48 h, except when measured at a more extended elbow angle (i.e., 160°) [34]. The acute rise in the shear modulus induced by the eccentric exercise was found to be independent of fluid accumulation, as evidenced by a comparison between the measured transverse relaxation time *T*_2_ and the shear modulus [34]. Nonetheless, there are studies reporting decreased resting muscle stiffness after fatiguing contractions of the knee [35] and trunk extensors [36].

Muscle shear modulus data have predominantly focused on arm and calf muscles, with limited research conducted on pennate thigh muscles such as the quadriceps femoris [37]. Furthermore, there is conflicting evidence regarding the impact of contractile activity on quadriceps muscle stiffness. Some studies have reported decreased or unchanged muscle stiffness [38], while others have demonstrated increased stiffness following strong contractions [39]. The accuracy of the relationship between the measured shear modulus and the mechanical status of the muscle is enhanced when the main axis of the US probe aligns parallel to the direction of the muscle fascicles. However, this accuracy is also influenced by the obliquity between the probe and the muscle fascicles within the plane of the US scan [6]. Therefore, to address some of these limitations, we conducted a preliminary study using SSI to assess changes in shear modulus of the vastus lateralis in response to variations in knee position and after a session of maximal isometric and isokinetic concentric (Conc) and eccentric (Ecc) contractions. Additionally, we investigated the response of the VL muscle to knee position using SSI and the relationship between VL shear modulus and submaximal knee extension torque. 

## 2. Material and Methods 

### 2.1. Participants

**Figure 1:**
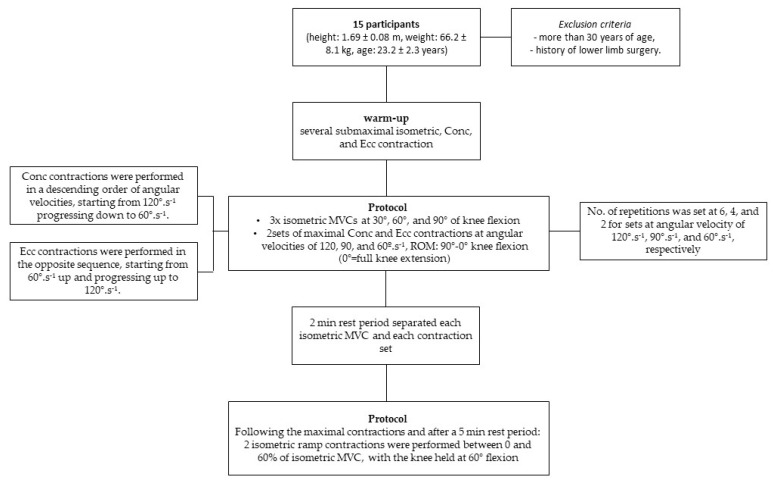
Protocol flowchart.

Fifteen young and active subjects (nine males; mean ± SD; height: 1.69 ± 0.08 m, weight: 66.2 ± 8.1 kg, age: 23.2 ± 2.3 years) volunteered for this study. The exclusion criteria were more than 30 years of age and having a history of lower limb surgery (Figure 1). All participants were fully informed about the purpose and procedures of the study and provided written informed consent. This study was approved by Ethics Council of the Faculty of Human Motricity (CEFMH 13-2013).

### 2.2. Protocol

Data collection was conducted in a single session and all muscle contractions were performed using an isokinetic dynamometer (Biodex System 3, Biodex Medical Systems, Shirley, NY, USA) (Figure 1). Participants were seated on the dynamometer chair, and the trunk was stabilized with the aid of chest and pelvis straps. The right leg was fixed to the dynamometer arm immediately above the malleoli, and the knee joint was aligned with the axis of the dynamometer arm. Participants remained seated on the dynamometer during the entire session. 

Following a warm-up consisting of several submaximal isometric, Conc, and Ecc contractions, participants performed a series of maximal contractions with the knee extensors. The protocol began with three repetitions of isometric MVCs at 30°, 60°, and 90° of knee flexion. This was followed by two sets of maximal Conc and Ecc contractions performed at angular velocities of 120, 90, and 60°·s^−1^, with a range of motion (ROM) between 90° and 0° of knee flexion (0° equating full knee extension). To match the contraction time between the different contraction velocities, the number of repetitions was set at 6, 4, and 2 for sets performed at angular velocities of 120°·s^−1^, 90°·s^−1^, and 60°·s^−1^, respectively. The isometric, Conc and Ecc contractions were performed in a randomized and counterbalanced order. The Conc contractions were performed in a descending order of angular velocities, starting from 120°·s^−1^ progressing down to 60°·s^−1^. The Ecc contractions were performed in the opposite sequence, starting from 60°·s^−1^ up and progressing up to 120°·s^−1^. A computer monitor provided feedback on the torque generated during the contractions and verbal encouragement was given during the efforts. A 2 min rest period separated each isometric MVC and each contraction set. 

Following the maximal contractions and after a 5 min rest period, two isometric ramp contractions were performed between 0 and 60% of isometric MVC, with the knee held at 60° flexion. Participants received visual feedback on the torque and time and were instructed to reach the target torque within approximately 10 s.

### 2.3. Region of Interest

**Figure 2:**
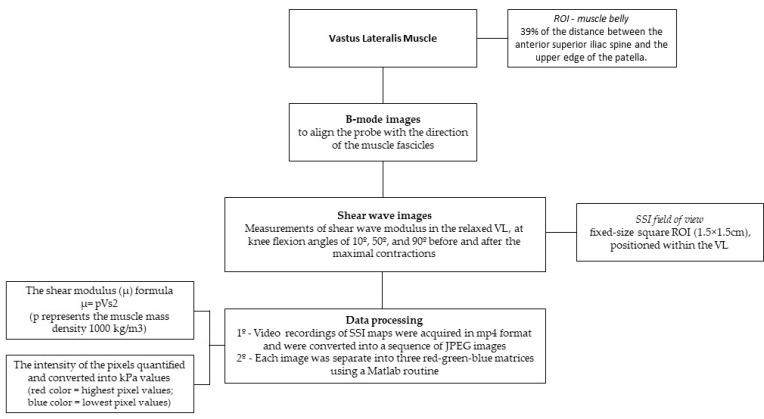
Imaging setting flowchart.

Figure 2 provides an overview of the imaging procedure. Firstly, for each participant, the region of interest was selected within a region of the muscle belly, centered at 39% of the distance between the anterior iliac spine and the upper edge of the patella.

### 2.4. Shear Wave Imaging

Shear wave imaging was performed using an Aixplorer US equipment (version 4.2; Supersonic Imagine, Aix-en-Provence, France) equipped with a 4–15 MHz linear transducer array (SuperLinear 15-4, Vermon, Tours, France) in shear wave elastography mode and musculoskeletal preset.

To scan the VL muscle, the probe was positioned at a location corresponding to 39% of the distance between the upper edge of the patella and the anterior superior iliac spine [40]. B-mode images were used to align the probe with the direction of the muscle fascicles. The optimal probe orientation was achieved when the hyperechoic images of the muscle fascicles were clearly visible and few muscle fascicles could be seen in their full length. Ultrasound gel was applied to ensure acoustic coupling. Once the anatomical location and probe orientation were set, skin landmarks were marked to ensure consistent probe placement over the same region of the VL during subsequent scans. Measurements of shear wave modulus in the relaxed VL were collected at knee flexion angles of 10°, 50°, and 90° before and after the maximal contractions. Participants were instructed to remain as relaxed as possible during these static measurements.

**Figure 3:**
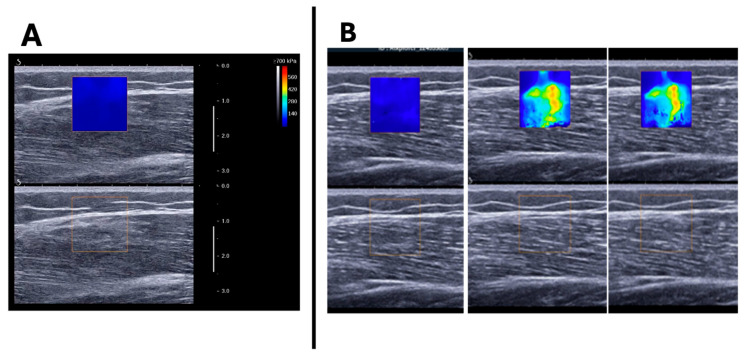
Example of image acquisition in longitudinal view: (**A**) image of vastus lateralis muscle and subcutaneous adipose tissue with and without the SII ROI (the ROI was reduced to 1.5 cm^2^ to avoid the superficial fascia and adipose tissue). (**B**) MP4 video acquired was converted into JPEG images and changes in color within the ROI were analyzed.

The SSI field of view was defined by a fixed-size square region of interest (ROI) measuring 1.5 × 1.5 cm, positioned within the VL, away from fibrous and adipose septa, and between the superficial and deep fascia (Figure 3). 

All scans were performed by the same operator, who has more than 10 years of experience. 

### 2.5. Data Processing

Video recordings of SSI maps were acquired in mp4 format. These videos were converted into a sequence of JPEG images. Next, each image was separate into three red–green–blue matrices using a Matlab routine (R2013a, The MathWorks Inc., Natick, MA, USA). The shear modulus (μ) is calculated assuming a linear elastic behavior of the tissues and using the following formula μ = pVs^2^, where p represents the muscle mass density (1000 kg/m^3^). The intensity of the pixels was quantified and converted into kPa values, with red color representing the highest pixel values and the lowest pixel values represented by the blue color. The upper limit of the shear modulus saturation was above 100 kPa [41].

### 2.6. Statistical Analysis

Two-way within-subjects ANOVAs were used to compare the shear modulus before and after knee extension contractions and between knee joint angles. This analysis was performed separately for each type of contraction (i.e., isometric, concentric, and eccentric) and for all contractions combined (baseline vs. end of the maximal contractions protocol). The sphericity assumption was tested with Mauchly’s test and, if this assumption was violated, the significance level was adjusted using the Greenhouse–Geiser correction. Polynomial contrasts were calculated for shear modulus values during ramp contractions using one-way ANOVA. The determination coefficient (R^2^) was obtained through linear regression analysis using the Pearson correlation coefficient. All statistical tests were conducted using SPSS software package, v.22 (SPSS Inc., Chicago, IL, USA). Data are presented as means ± SD. Statistical significance was accepted at *p* < 0.05.

## 3. Results

The VL shear modulus could not be measured during ramp contractions in one participant. Therefore, all analyses were conducted with data from 15 participants. 

At baseline, the relaxed VL shear modulus was 5.06 ± 1.48, 5.68 ± 2.08, and 9.40 ± 4.10 kPa at 10, 50, and 90° knee flexion, respectively. At the end of the entire protocol of maximal contractions, the relaxed VL shear modulus increased to 5.87 ± 2.83, 7.05 ± 3.65, and 10.95 ± 2.92 kPa at 10, 50, and 90 degrees of knee flexion, respectively. Two-way ANOVA revealed a significant main effect of maximal contractile activity [pre vs. post: F_(1,14)_ = 8.122; *p* = 0.013, η^2^ = 0.367] and knee angle [F_(2,28)_ = 43.467; *p* < 0.001, η2 = 0.756] on relaxed VL shear modulus, but there was no interaction effect between these two factors [F_(2,28)_ = 0.160; *p* = 0.853, η2 = 0.098] (Table 1; Figure 3A). Compared to baseline, the shear modulus increased after isometric [F_(2,28)_ = 9.354; *p* = 0.009, η^2^ = 0.401] and Ecc [F_(2,28)_ = 6.512; *p* = 0.023, η^2^ = 0.317] contractions but not after Conc contractions [F_(2,28)_ = 4.358; *p* = 0.056, η^2^ = 0.237]. There were no significant interaction effects between time and knee angle for any of the contraction types (Table 1). 

**Figure 4:**
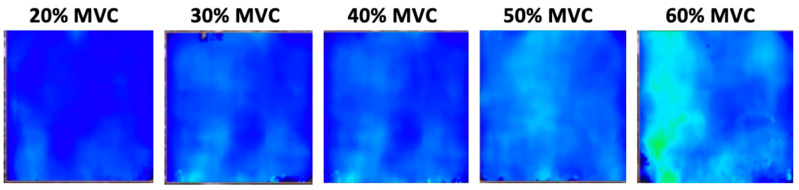
Typical shear wave elastograms during ramp isometric contractions of increasing intensity. MVC, maximal voluntary contraction. During ramp contraction, as the percentage of maximal voluntary contraction (MVC) increases, the elastogram color shifts from blue to green, indicating an increase in shear modulus and muscle stiffness with higher contraction intensity.

**Figure 5:**
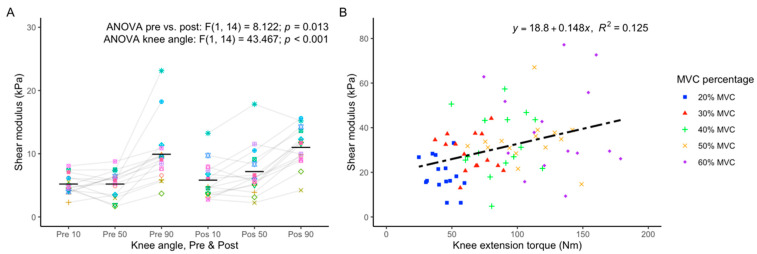
(**A**) Values of VL shear modulus at 10°, 50°, and 90° of knee flexion angle at the beginning (Pre) and end (Post) of the series of maximal isometric, Conc, and Ecc contractions. Individual data points are shown, with mean values indicated by the horizontal bars. ANOVA outputs are shown at the top of the plot. (**B**) Individual VL shear modulus during isometric knee extension at different levels of contraction. The linear regression equation relating VL shear modulus to knee extension torque and associated R^2^ value are shown at the top of the plot. VL_ Vastus Lateralis; MCV: Maximal Voluntary Contraction.

During ramp contractions, the VL shear modulus increased linearly with torque production (Figure 4). The R^2^, calculated from the individual linear regressions between muscle torque and VL shear modulus (excluding resting values), had a mean value of 0.77 ± 0.33 (range: 0.27–0.99). However, pooling the individual data together, a large variation in the VL shear modulus changes during isometric contraction becomes apparent, particularly at higher contraction intensities (Figure 5B). Polynomial contrasts revealed a significant linear effect between the VL shear modulus and the percentage of isometric MVC [F_(1,14)_ = 37.934; *p* < 0.001], while the quadratic contrast was not significant [F_(1,14)_ = 0.512; *p* = 0.482]. This suggests that, at least up to 60% MVC, the VL shear modulus continues to increase linearly without levelling off. 

## 4. Discussion

In this study, three main observations were made regarding changes in the VL shear modulus. Firstly, we found an increase in the relaxed VL shear modulus with knee flexion, particularly between 50° and 90°. Secondly, a linear relationship was observed between the degree of isometric contraction and VL shear modulus. Lastly, there is an overall increase in VL shear modulus after performing a series of maximal isometric, Conc, and Ecc contractions. In general, these observations agree with other reports [29,33,42]. 

Several studies have reported a relationship between relaxed muscle length and the shear modulus for various lower limb muscles [5,29,43,44,45]. Nordez et al. [45] used transient US elastography to establish a linear relationship between the stiffness of the medial gastrocnemius and ankle joint angle, as well as passive ankle torque. The study reported a 2.6-fold increase in medial gastrocnemius stiffness when the ankle moved passively along an 80° arc (from 40° plantar flexion to 40° of dorsiflexion) [45]. Le Sant et al. (2015) also found a strong relationship between the shear modulus of the hamstrings and the position of the knee and hip joints [29]. However, the increase in shear modulus observed in the passively stretched hamstrings, ranging between 9.7 and 13.2 kPa, appeared to be greater than the increase in VL stiffness recorded in our study. The exact relationship between relaxed muscle stiffness and joint position may depend on the morphological characteristics of the muscle and joints [5,42]. In the case of the VL, we observed that the increase in the shear modulus with knee flexion was not linear; it was larger when the knee was bent from 50° to 90° than between 10° and 50°. This observation agrees with data from other pennate muscles and may be partially attributed to a diminished obliquity of the muscle fascicles relative to probe orientation [6].

The main purpose of this study was to evaluate the acute effect of intense muscle activity on relaxed muscle stiffness using SSI. Our results clearly demonstrate a significant increase in muscle stiffness following intense contractile activity, which is consistent with previous studies [33,34,39,46] but in disagreement with other reports [33,38,47]. Lacourpaille et al. [34] reported an elevation in passive stiffness of the biceps brachii one hour after three sets of ten maximal isokinetic eccentric contractions [23]. Similarly, Akagi et al. [48] observed increased passive stiffness of the triceps brachii immediately after five sets of eight repetitions of a dumbbell extension exercise performed at 80% of one repetition maximum [48]. Notably, the increase in passive stiffness observed in these studies was unrelated to the degree of muscle swelling. These results contrast with the effect of fatiguing submaximal muscle activity on passive muscle stiffness [33,49]. Nordez et al. [33] reported a decrease in muscle stiffness of the medial gastrocnemius immediately after a fatiguing isometric contraction of the ankle plantar flexors performed at 40% MVC [33]. Furthermore, Andonian et al. [49] demonstrated a significant reduction in passive stiffness of the superficial heads of the quadriceps femoris (i.e., the VL, VM, and RF) after completion of a mountain ultra-marathon [49]. Recent studies also report substantial reduction in relaxed vastus lateralis [35] and multifidus muscles after fatiguing isometric contractions [36]. Therefore, the impact of muscle activity on muscle stiffness seems to depend on the relative intensity, the duration, and possible the type of the contractions. In our study, the acute increase in the relaxed VL stiffness was more evident after isometric and Ecc maximal contractions than after maximal Conc contractions. However, this observation should be interpreted with caution since all types of contractions were performed within the same session. In a previous study, we reported increased VL stiffness after fifteen weeks of concentric and eccentric strength training [12], corroborating the results of the present study.

Supersonic shear imaging has been proven valuable for determining the level of contraction of a single muscle by establishing the relationship between the muscle force (or torque) output and its stiffness [13,31,50]. This use of SSI is particularly important for studying force sharing between synergistic muscles in various contexts [51,52]. In our study, we observed a highly precise individual relationship between the amount of isometric knee extension torque and the VL stiffness. Previous research on hand muscles has demonstrated that the relationship between muscle stiffness and force output exhibits higher accuracy compared to the relationship between surface electromyography and torque [52]. However, in the present study, the mean coefficient of determination between VL stiffness and knee extension torque was 0.77, which is lower than 0.98, the value reported for hand muscles [52]. Furthermore, we observed a considerable variation in the coefficient of determination across our participants, with larger scattering of VL shear modulus values with increased knee extension torque. The underlying reasons for such variability cannot be fully determined, but it may be related to the greater difficulty in constraining the action of knee extensors compared to muscles like the first dorsal interosseus or the abductor digiti minimal [52] or to differences in motor unit recruitment between the hand muscles and the VL. Furthermore, the ramp contractions in our study were performed at the end of the session, following the maximal contractions, potentially influencing the results due to muscle fatigue [51].

## 5. Limitations

This study has several limitations due to its preliminary nature. Firstly, the study was conducted in a single session with a non-randomized order of maximal isometric, concentric, and eccentric contractions, as well as ramp contractions. Therefore, the cumulative effect of these maximal contractions cannot be distinguished in the magnitude of the responses recorded from pre to post measurements. Although it is a pilot study, the small sample size is another limitation. Therefore, caution should be taken in drawing definite conclusions based on these findings. Additionally, data collection was limited to immediate post-session measurements, and long-term recovery was not monitored. The size of the ROI used was standardized but only probed a small region of the VL, which may not be representative of the entire muscle.

## 6. Conclusions

In conclusion, using SSI, this study revealed non-linear increases in the passive stiffness of the vastus lateralis with knee flexion, as well as acute increases in this muscle stiffness following maximal isometric and eccentric contractions. However, the impact of concentric contractions on vastus lateralis passive stiffness was less conclusive. Furthermore, a reasonable accurate relationship between the amount of isometric torque and vastus lateralis stiffness was demonstrated. These findings contribute to the existing body of research demonstrating the potential of SSI in studying muscle function in humans. Future research should investigate how different types of contraction affect muscle stiffness and explain the individual variation in the contraction intensity–muscle stiffness relationship.

## Figures and Tables

**Table 1:** Two-way within-subjects ANOVA results for the effect of maximal contractions on VL relaxed shear modulus.

Test	Factor Knee Angle			Factor Pre–Post			Interaction Effect		
	Degrees of Freedom Factor; Error	F-Ratio	*p*-Value	Degrees of Freedom Factor; Error	F-Ratio	*p*-Value	Degrees of Freedom Factor; Error	F-Ratio	*p*-Value
Pre vs. Post all contractions	2, 28	43.467	<0.001	1, 14	8.122	0.013	2, 28	0.160	0.853
Pre vs. Post isometric	2, 28	31.067	<0.001	1, 14	9.356	0.009	2, 28	1.67	0.847
Pre vs. Post concentric	2, 28	43.675	<0.001	1, 14	4.358	0.056	2, 28	1.516	0.237
Pre vs. Post eccentric	2, 28	25.612	<0.001	1, 14	6.512	0.023	2, 28	0.963	0.394

## Data Availability

Materials described in the manuscript, including all relevant raw data, will be freely available upon request to the corresponding author, to any scientist wishing to use them for non-commercial purposes, without breaching participant confidentiality.

## Abbreviations

US: ultrasound; SSI: supersonic shear imaging; VL: vastus lateralis; Conc: concentric; Ecc: eccentric; MVC: maximal voluntary contraction; ROI: region of interest.
